# The underlying mechanism of Guillain-Barré syndrome in a young patient suffered from Japanese encephalitis virus infection: a case report

**DOI:** 10.1186/s12985-022-01870-7

**Published:** 2022-09-01

**Authors:** Sheng Liu, Jinyong Wang, Jun Yang, Ying Wen

**Affiliations:** 1 Department of Infectious Diseases, The First Affiliated Hospital of China Medical University, No. 155, Nanjing North Street, Heping District, Shenyang, Liaoning Province China; 2 Neurology Department, The First Affiliated Hospital of China Medical University, Shenyang, Liaoning Province China

**Keywords:** Guillain-Barré syndrome, Japanese encephalitis virus, Vaccination, Pathogenesis

## Abstract

**Background:**

The presentation of Guillain–Barré syndrome (GBS) caused by Japanese encephalitis virus (JEV) is uncommon, although clusters of GBS cases were observed in China in 2018. The underlying mechanism is unclear, particularly in individuals vaccinated against Japanese encephalitis in childhood.

**Case presentation:**

We report a patient with acute flaccid paralysis involving four extremities and respiratory muscles, while magnetic resonance imaging of the brain and spine were standard. Electrophysiological examination displayed slowed motor nerve conduction speed and reduced evoked velocity amplitude. GBS was finally considered which was related to JEV infection verified by positive anti-JEV immunoglobulin M antibody and positive immunoglobulin G antibody in the serum. Unfortunately, the patient refused intravenous immunoglobulin and declined the use of mechanical ventilation again. He voluntarily withdrew from the hospital and died on the 36th day after the onset of illness. We also performed a review of previously reported related cases and discussed the underlying mechanism.

**Conclusion:**

JEV infection-associated GBS is unusual. We should pay attention to the atypical manifestations of JEV infection and explore possible pathogenesis in particular individuals.

## Background

The incidence rate of Japanese encephalitis (JE) is 30,000–50,000 cases yearly [1]. JE is a mosquito-borne zoonotic disease mainly occurring in eastern and southern Asia, caused by the Japanese encephalitis virus (JEV). The prevalence of JE has decreased in China due to vaccination programs. JEV genotype 1 is currently circulating in China. The typical clinical manifestation includes fever, headache, vomiting, neurological symptoms, coma, convulsions, and respiratory failure [2]. Most cases with JEV-associated acute flaccid paralysis (AFP) met the case definition of Guillain–Barré syndrome (GBS), while a small number of cases were thought to have myelitis [3, 4]. Herein we report a young man with JEV infection who developed GBS after several days of fever. The underlying mechanism warrants further study.

## Case presentation

**Fig. 1:**
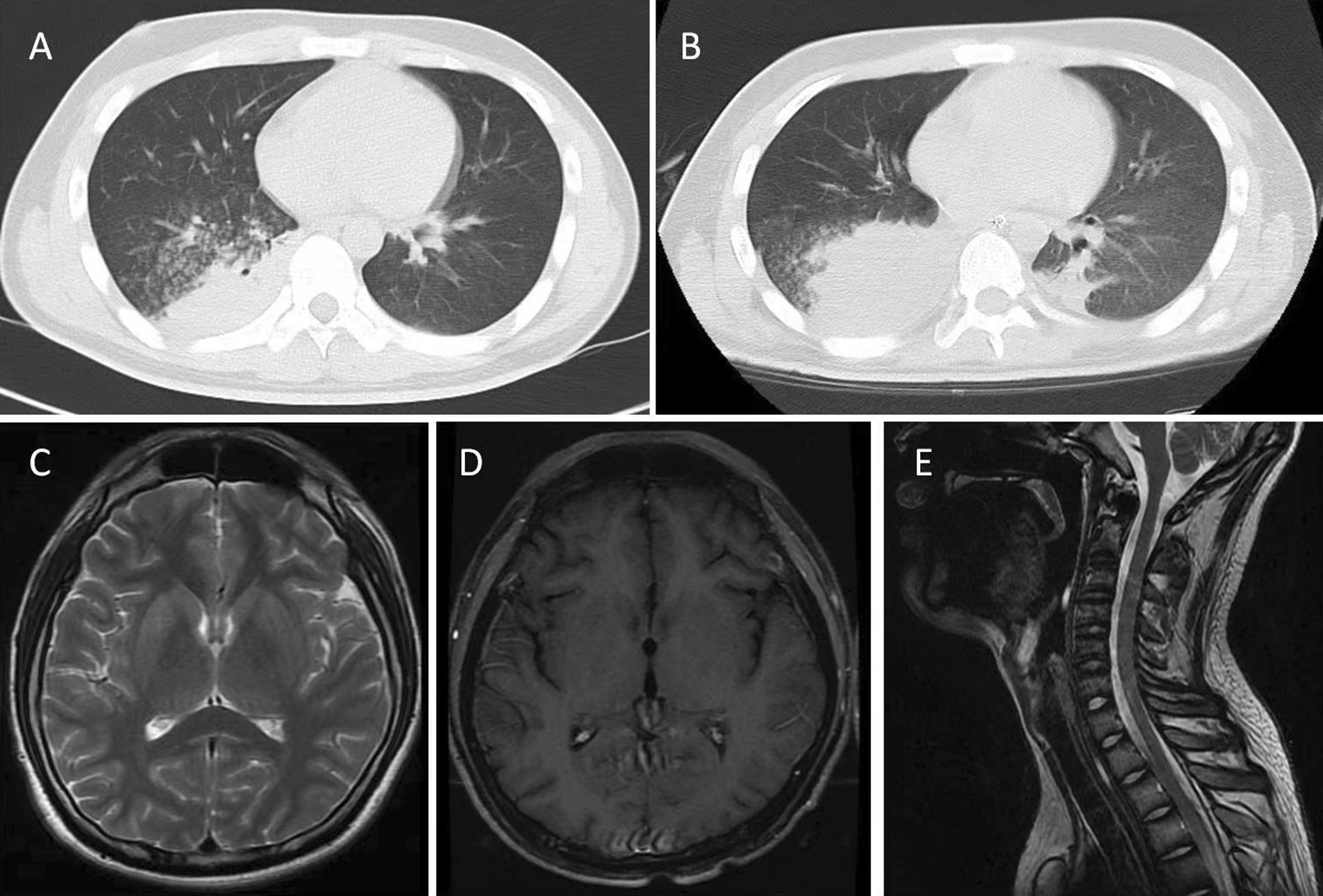
The radiographs in this case. **A** At 19th day of illness onset, lung CT indicated consolidation in the right lung. **B** At 24th day of illness onset, lung CT indicated aggravated consolidation in the lower lobe of bilateral lung adjacent to the pleura, mainly in the right lung. **C** At 5th day of illness onset, brain MRI (T2 weighted image) was normal. **D** At 24th day of illness onset, enhanced brain MRI was normal. **E** At 24th day of illness onset, spine MRI (T2 weighted image) was normal

A young man aged 18 years first developed muscle weakness of the bilateral lower limbs, followed by fever, dizziness and muscle weakness of the neck and bilateral upper limbs. He had no gastrointestinal or respiratory symptoms. On the 5th day after illness onset (on October 3, 2018), he felt dyspnea without convulsions or impaired consciousness and was referred to the emergency room of our hospital. He was intubated and invasive mechanical ventilation was employed due to dyspnea progression. The brain's magnetic resonance imaging (MRI) was normal (Fig. 1C). Blood tests revealed elevated white blood cell counts (11.79 × 10^9^/L) and neutrophil count (9.56 × 10^9^/L). Anti-JEV immunoglobulin (Ig) M antibody (EEB-IgM, Enzyme immunoassay test kit, Shanghai B&C Biological Technology Company) was positive in his serum. Unexpectedly, a high level anti-JEV IgG antibody (JE detect ^TM^IgG, ELISA, InBios) was also detected in his serum. Polymerase chain reaction (PCR) of serum was negative for JEV RNA. PCR was also negative for other viruses such as Zika, Dengue, West Nile, Forest encephalitis, Coxsackie, poliovirus, Echovirus, Enterovirus, and herpesviruses. The cerebrospinal fluid (CSF) showed elevated leukocyte counts (49 × 10^6^/L, lymphocytic pleocytosis) and protein level (814 mg/L, normal range: 100–600 mg/L). Indian ink staining, acid-fast staining and bacterial and fungal culture of the CSF sample were all negative. Unfortunately, no CSF sample was available for anti-JEV IgM and JEVRNA testing. The patient was from a rural area in the Liaoning province of China, where a JEV outbreak occurred in the summer of 2018. He was vaccinated against JE in childhood at eight months and two years of age. He was medicated with methylprednisolone (500 mg per day) followed by tapering.

Ten days later, his condition partly improved. He refused to continue invasive ventilation though he still had respiratory disturbance with an elevated PaCO_2_of 58.0 mmHg and decreased PaO_2_of 72.0 mmHg (on intranasal oxygen therapy of 10 L/min). Despite lucidity, proper cognition, and functional bladder and bowel, he had marked disturbances in coughing, articulation, and swallowing with absence of pharyngeal reflex and disabled soft palate movement, indicating injured cranial nerves (bulbar paralysis). He performed pectoral type breathing without any thoracic breathing, suggesting respiratory myoparalysis. Examination revealed weakened muscle power in the left upper limb (grade 4/5), right upper limb (grade 2/5), and bilateral lower limbs (grade 2/5), which were improved compared to day after illness onset. The muscle tone of his four extremities was decreased. Deep tendon reflexes were decreased without pathological reflex and sensation disturbance.

Auscultation revealed a purring sound in bilateral lungs. Lung computed tomography (CT) indicated consolidation in the lower lobe of the right lung (Fig. 1A). The blood test was negative for the antibodies of Epstein–Barr virus, herpes simplex virus, varicella-zoster virus, cytomegalovirus and human immunodeficiency virus. On the 24th day after illness onset, repeated MRI of the brain and spine appeared normal (Fig. 1D, E).The patient refused a second lumbar puncture. The assay for anti-gangliosides antibodies was unavailable at our hospital. On the 27th day after illness onset, electrophysiology examination revealed abnormal findings including prolonged latency of H reflex in bilateral tibial nerves, reduced amplitude of evoked velocity in the right median nerve, and slowed motor conduction in the right tibial nerve, while the conduction of the sensory nerves was normal. JEV infection-associated GBS was considered, which agreed with Brighton criteria level 1. The patient declined intravenous Ig and mechanical ventilation. Despite applying of broad-spectrum antibiotics and sputum clearance with bronchoscopy, he developed progressive dyspnea, increased carbon dioxide retention, and respiratory acidosis (PaCO_2_, 120 mmHg; PH, 7.185). Lung CT indicated aggravated consolidation in the lower lobe of bilateral lung, mainly in the right lung (Fig. 1B). He still refused mechanical ventilation and tracheotomy. He voluntarily withdrew from the hospital and died on the 36th day after illness onset.

We prepared a literature review of a total of 85 cases of JEV-associated AFP reported previously (Table 1), 73 cases were considered as GBS. There were 4 cases with a reported history of previous JE vaccination.

**Table 1:** Clinical features in patients with acute flaccid paralysis caused by Japanese encephalitis virus

Case numbers	Reference number/year of publication	Age (year)	Gender (M/F)	Area of report	Clinical presentations	Encephalitis	Brain MRI	JEV detectetion in serum/CSF	JE vaccination	Treatment	Outcome D/S/N
1	[3]/2007	22	1/0	Taiwan	AFP	No	Normal	JEV-IgM+/+	Yes	GC + MV	S
21	[5]/1994	6–58	18/3	India	GBS	No	NM	JEV-IgM+/+	NM	GC or MV	4/15/2
1	[6]/2014	23	1/0	China	GBS	No	Normal	JEV-IgM+/+	No	IG + GC	S
47	[11]/2020	*59 (24–63)	26/21	China	GBS	39	NM	JEV-IgM+/+	Yes(2)	IG(28) + GC(47) + MV(44)	S
12	[14]/1998	3–15	9/3	Vietnam	AFP including GBS(1)	Yes (4)	NM	JEV-IgM+/+	NM	No MV	S
1	[15]/2015	14	1/0	India	GBS	Yes	Abnormal	JEV-IgM+/+	NM	IG + MV	S
1	[16]/2021	43	0/1	China	GBS	Yes	Abnormal	JEV-IgM+/+	NM	IG + GC + MV + PAIA	S
1	PR	18	1/0	China	GBS	No	Normal	JEV-IgM+/ND	Yes	GC + MV	D

*GBS* Guillain–Barre syndrome; *AFP* acute flaccid paralysis; *NM* not mentioned; *PR* present report; *D* died; *S* survived; *N* no follow up; *ND* not done; *IG* immunoglobulins; *GC* glucocorticoid; *MV* mechanical ventilator; *PAIA* protein A immunoadsorption

*Median age (IQR)

## Discussion and conclusion

GBS is generally regarded as a post-infection immune-mediated disease. JEV infection is an important etiology of GBS in endemic regions [5]. Most patients with JEV-associated GBS did not receive the JE vaccination previously [6].Our patient is similar to the case reported by Chung, et al [3] of a patient who had also received JE vaccination in childhood. The high ratio of IgG to IgM is a distinction between rapid seroconversion of IgM and IgG due to acute infections and IgG antibodies derived from previous vaccine viruses. All available vaccines belong to the JEV genotype 3 strain. A partial cross-protection between JEV genotype 1 and genotype 3 strain [1, 7] and the inferior protective efficacy due to inadequate neutralizing antibody levels probably explain why JE vaccination could not provide complete protection.

Liaoning province is located in northeast of China, where neither Zika nor Dengue virus [8, 9] are endemic. Although Liaoning province had a low prevalence of JE [10], there was an outbreak of 69 cases with a 30.4% fatality rate in the summer of 2018, which was very similar to the prevalence of JEV infection in the north of Ningxia in 2018 [11]. JEV genotype 1 was the pathogen in that outbreak, and no cases were reported in children, indicating that the JEV vaccine was still effective. The chance of JEV detection by PCR in the blood and CSF is extremely low [4, 8].Our patient was confirmed with JEV infection by positive anti-JEV IgM antibody in serum, a sensitive, specific, and early indicator of JEV infection. His characteristic clinical manifestation included weakness of the four extremities and respiratory myoparalysis accompanied by the involvement of several cranial nerves. Electrophysiology examination suggested decreased evoked potential amplitude and slowed conduction speed of motor nerves. Acute disseminated encephalomyelitis and acute transverse myelitis were excluded based on normal brain and spinal MRI images [4, 12]. Glucocorticoid-induced myopathy was also ruled out as his muscle paralysis preceded glucocorticoid application [13]. We must also differentiate between anterior horn cell myelitis [3, 14] and GBS [5, 15, 16]. Viral myelitis tends to present with a fever and a moderate pleocytosis in the CSF, while GBS typically presents a prodromal infection 1–2 weeks prior and an albumin cytologic dissociation in the CSF. However, anterior horn cell myelitis was not supported by the normal MRI of the spinal cord and slowed motor nerves conduction speed. Therefore, GBS was finally considered. The mortality of GBS is 2–10% [17]. GBS was involved in the pathogenesis of humoral and cellular immunity [16]. The underlying mechanism for the clusters of GBS cases in 2018 was unclear [11]. Molecular mimicry, antiglycolipid autoantibody, and immune complexes were implicated in the pathogenesis of GBS, which caused a kind of demyelinating neuropathy or axonal neuropathy [18, 19]. Although this patient received two doses of JEV vaccine in childhood, another booster vaccine should be provided by reason of the reduced protective antibody levels over time or primary “low-responders” [20]. Moreover, the presence of high levels of anti-JEV IgG in early serum provided the possibility that antibody-dependent enhancement (ADE) between pre-existing antibody targeting genotype 3-derived JE vaccine and subsequent genotype 1 virus infection may play a role in the development of GBS [21].

In summary, JE combined with GBS shows an increasing trend in recent years. Rapid diagnosis and early application of intravenous Ig or plasma exchange will be beneficial for GBS while glucocorticoids are not recommended [17, 21, 22]. JEV genotype 1 is currently circulating in China [23]. The longevity and titers of protective antibody responses induced by the JE vaccine depend on the type of vaccine, number of doses, and the immune status of hosts. Up to now, there is no standardized protocol for a neutralization test. A commercial kit for neutralizing antibody detection is unavailable. Therefore, another booster dose should be recommended approximately a decade after the first booster immunization. The protective role of genotype 3-derived JE vaccine in preventing genotype 1 virus infection needs additional attention.

## Data Availability

All data generated or analyzed during this study are included in this published article.
